# Extrapolation beyond the end of trials to estimate long term survival and cost effectiveness

**DOI:** 10.1136/bmjmed-2021-000094

**Published:** 2022-03-10

**Authors:** Nicholas R Latimer, Amanda I Adler

**Affiliations:** 1 School of Health and Related Research, University of Sheffield, Sheffield, UK; 2 Diabetes Trials Unit, University of Oxford, Oxford, UK

**Keywords:** economics, health policy, statistics

**Key messages**

- Extrapolation beyond time periods studied in clinical trials is usually necessary to estimate long term effects of treatments
- Many statistical survival models can be used to extrapolate data, but these can have widely varying results, which affects estimated clinical effectiveness and cost effectiveness
- The choice of survival model and credibility of the extrapolations should be inspected carefully when making policy decisions that inform the allocation of healthcare resources

This paper explains the importance of extrapolating beyond the end of trials to estimate the long term benefits associated with new treatments, why this is done, and the limitations of various approaches.

## Introduction

Policy makers worldwide use economic evaluation to inform decisions when allocating limited healthcare resources. A critical part of this evaluation involves accurately estimating long term effects of treatments. Yet, evidence is usually from clinical trials of short duration. Rarely do all participants encounter the clinical event of interest by the trial’s end. When people might benefit from a long term treatment, health technology assessment agencies recommend that economic evaluations extrapolate beyond the trial period to estimate lifetime benefits.^1 2^ This kind of evaluation is common for people with cancer, when effective treatments delay disease progression and improve survival.

### Use of survival modelling: rationale

To make funding decisions, health technology assessment agencies rely on accurate estimates of the benefits and costs of new treatments compared with existing treatments. For treatments that improve survival, accurate estimates of survival benefits are crucial. Policy makers use estimates of mean (average) survival rather than median survival, taking into account the probability of death over a lifetime across all patients with the disease. This mean is represented by the area under survival curves that plot the proportion of patients alive over time by treatment.

**Figure 1:**
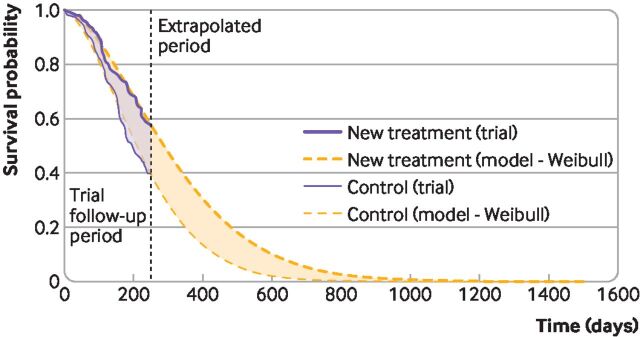
Survival modelling to extrapolate beyond the trial—mean survival restricted to the trial period, and extrapolated

In figure 1, the purple area represents a mean survival benefit associated with an experimental compared with a control treatment, but this benefit is a restricted mean, limited to the trial period. The curves separate early, and remain separated at the end of the trial, so it is reasonable to expect that benefits would continue to accrue beyond the trial’s end. The orange smooth curves represent survival models fitted to the trial data and extrapolated beyond the trial. The area between the orange curves estimates the mean lifetime survival benefit associated with the experimental treatment. This area is much larger than the purple area, and is relevant for economic evaluation.

### Description of survival models

Survival models extrapolate beyond the trial. They typically have a parametric specification, which means that they rely on an assumed distribution of probabilities of, for example, death over time, which is defined by a set of parameters such as shape and scale. The chosen parametric model is fitted to the observed trial survival data, and values estimated for each parameter. The model is then used to generate survival probabilities beyond the trial period to predict what would have happened had the trial continued until everyone died.

**Figure 2:**
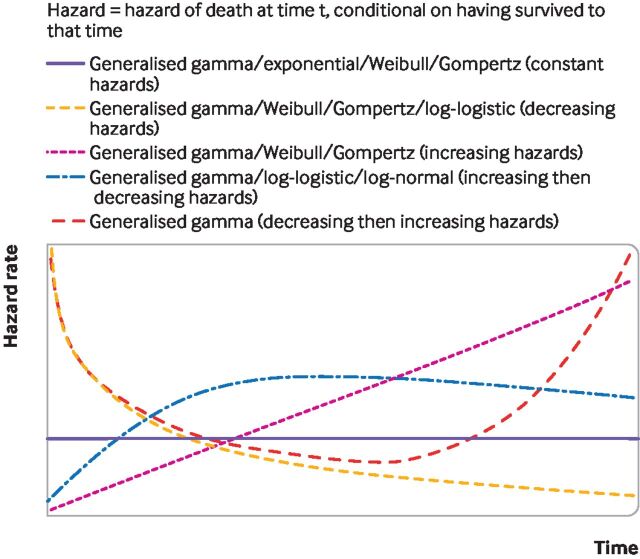
Survival modelling to extrapolate beyond the trial—hazard shapes associated with standard parametric survival models

In health technology assessments, a set of standard models typically include: exponential, Weibull, Gompertz, log-logistic, log-normal, and generalised gamma models.^3^ Each survival model involves different assumptions about the shape of the hazard function—that is, the risk over time of the event of interest,—which is usually death. Figure 2 shows the hazard function shapes assumed when using standard parametric models; over time these can stay the same, increase, decrease, or have one turning point (that is, the hazard increases then decreases, or decreases then increases).

### Selecting a model

**Figure 3:**
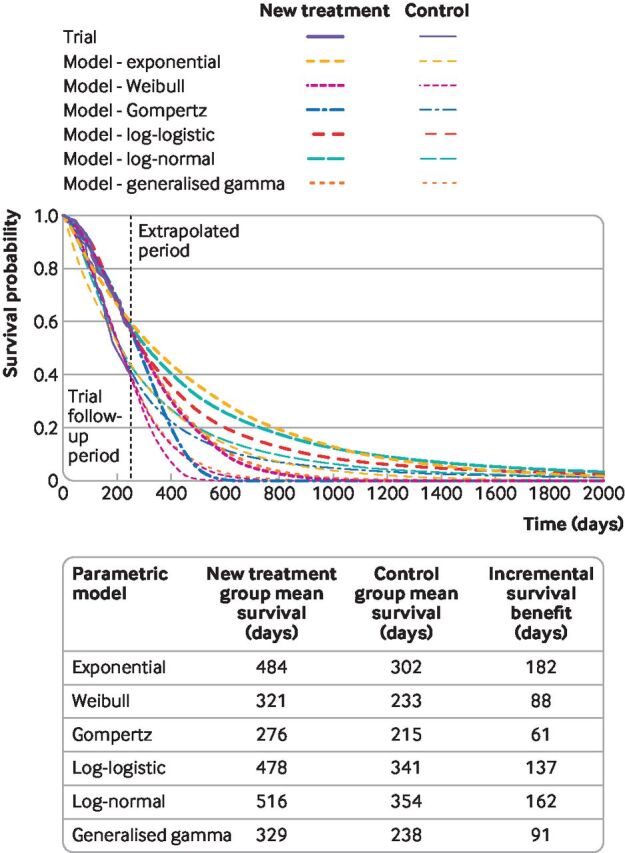
Survival modelling to extrapolate beyond the trial—a variety of standard parametric models fitted to the same data

Extrapolating survival curves predicts the unknown. No one can know which models most accurately predict survival—although it might be possible to determine which models produce extrapolations that are plausible. Different models often result in substantially different estimates of survival and cost effectiveness.^4^ Figure 3 shows a range of survival models fitted to the same data. While all the parametric models seem to fit the observed trial data well, they predict large differences in longer term and mean survival. The more immature the trial data, the more likely the long term predictions will differ. Model choice affects estimated treatment benefits and, consequently, cost effectiveness.

To choose clinically plausible survival models, modellers must assess fit to the trial data, but also, crucially, assess the credibility of the extrapolations.^4 5^ This approach involves considering external data sources with longer term data such as other trials, disease registries, and general population mortality rates. Biological plausibility, pharmacological mechanisms, and clinical opinion should also be considered. Although identifying a single best model might not be possible, this approach ensures that policy makers use credible models.

### Limitations of standard survival models

**Figure 4:**
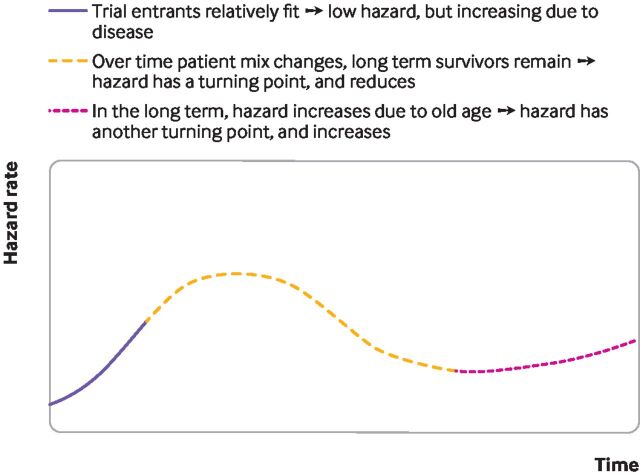
Survival modelling to extrapolate beyond the trial—a hypothesised, realistic hazard function

Standard parametric survival models have limitations. They might rely on hazard functions with implausible shapes (figure 2), and might neither fit the data well nor provide credible extrapolations. As illustrated in figure 3, the implications of choosing the wrong survival model are serious, because the choice of model affects survival predictions. Figure 4 illustrates a hypothetical hazard function of death from a cancer. No standard parametric models could capture the shape of this function, although more complex survival models can, such as flexible parametric models, fractional polynomials, piecewise models, or mixture cure models.

Flexible parametric models (such as restricted cubic spline models) segment the survival curve into portions, using knots to model hazard functions that have many turning points.^6^ However, flexible parametric models will not generate turning points beyond the period of observed trial data unless modellers use external information, which they rarely do, such as longer term hazard rates from registry data. Indeed, while flexible parametric models are likely to fit the data well, beyond the data they reduce to standard Weibull, log-normal, or log-logistic models (therefore assuming that a transformation of the survival function is a linear function of log-time), and might generate implausible extrapolations. In figure 4, if the trial were short and ended in the period where the hazard function is rising, a flexible parametric model would extrapolate that rising hazard, based on the observed segment of data.

An alternative option is to use fractional polynomials to model a hazard function with a complex shape, placing no restrictions on the hazard and survival functions beyond the period of observed data. However, while these models might fit the observed data well, the lack of restrictions on the extrapolation can lead to implausible predictions.^7^ Other options include piecewise models, where separate survival models are fitted to defined portions of the observed survival data using cut-off points. The extrapolation is based on the model fitted to the final observed period. Piecewise models can be sensitive to the choice of cut-off points, and lead to extrapolations based on the last portion of data where numbers of trial participants and numbers of deaths among these participants are often low.^8^ Generalised additive models and dynamic survival models have recently been suggested as potentially valuable novel approaches for modelling and extrapolating survival data.^7^

Mixture cure models can capture complex hazard functions because they predict survival separately for cured and uncured patients,^9^ and estimate a cure fraction—that is, the proportion of patients who would be cured. Predicting survival for cured and uncured patients separately could result in a model that generates credible extrapolations. However, a key issue that is difficult—or perhaps impossible—is to estimate a cure fraction reliably based on short term data. When the cure fraction is estimated inaccurately, cure models can result in poor survival predictions.

### Extrapolation in practice

Decision makers, such as those on committees of the National Institute for Health and Care Excellence (NICE), discuss, document, and assess the approaches that pharmaceutical companies use to predict long term survival. Often the approach has a large impact on cost effectiveness estimates (box 1). Typically, NICE reviews appraisals three years after the initial recommendation, and some drugs are placed in the Cancer Drugs Fund, providing an opportunity for checking extrapolations once longer term data are available, often from the key trial. However, while drugs in the Cancer Drugs Fund undergo rigorous reappraisal, other reviews are rarely done comprehensively, leaving extrapolations unchecked.

Box 1 **Impact of survival modelling in technology appraisals by the National Institute for Health and Care Excellence (NICE)**

When NICE appraised pembrolizumab for untreated, advanced oesophageal and gastro-oesophageal junction cancer, the appraisal committee identified four approaches to survival modelling that it considered to be credible.^10^ These approaches were a log-logistic piecewise model, a log- logistic piecewise model incorporating an assumed waning of the treatment effect over time, a log-logistic model not fitted using a piecewise approach, and a generalised gamma piecewise model. The incremental gains in quality adjusted life years (QALYs) associated with pembrolizumab ranged from 0.50 to 1.07 QALYs per person over a lifetime, with the estimated cost per incremental QALY doubling between the most and least optimistic analysis.^11^

When NICE appraised tisagenlecleucel (a chimeric antigen receptor T cell treatment) for relapsed or refractory, diffuse, large B cell, acute lymphoblastic leukaemia, the committee acknowledged that survival was a key uncertainty, considered cure possible, and discussed several mixture cure models. Cure fractions varied by 35 percentage points depending on the model, with cost effectiveness estimates that varied from potentially acceptable to unacceptable.^12^ The committee accepted using a mixture cure model based on clinical experts suggesting that some patients could be cured. However, the committee preferred a model that estimated a lower cure fraction than that estimated by the manufacturer’s preferred model, because the manufacturer’s model predicted a cure fraction that was higher than the proportion of patients who remained event-free in the tisangenlecleucel trials. Tisagenlecleucel was recommended for use in the Cancer Drugs Fund to allow the trial to accrue more data on overall survival before making a final decision on its routine use in the NHS.^12^

## Conclusions

When treatments make people live longer, it is important to extrapolate beyond the end of clinical trials to estimate mean survival gains and cost effectiveness over a period longer than the trial. Several survival models are available, and these result in widely varying estimates. To choose a model, researchers should consider a model’s fit to the observed trial survival data, and the credibility of predictions beyond the trial. More complex models could, but do not necessarily, result in better extrapolations. To inform decision making, survival models must be scrutinised while considering a range of plausible models and their impact on cost effectiveness. Analysts should follow recommended processes, report analyses clearly, justify chosen models by describing why and how the models have been selected, detail how well models fit the observed data, and describe what the models predict about hazards and survival.^4 8^ This approach provides decision makers with the reassurance needed to make decisions when allocating healthcare resources.

## Data Availability

No patient level data were used for this article. The survival curves illustrated in figures 1 and 3 were fitted to data simulated by NRL. Please email the corresponding author to request access to the simulated data.
